# Late Presentation of Peritonitis Post Jejunal Perforation Due to Flank Stab Wound

**DOI:** 10.7759/cureus.31351

**Published:** 2022-11-10

**Authors:** Fatima H Al Saeed, Sarah H AlShawaf, Hassan N Al Dhneem, Abdullah A AlZahid, Dunya Alfaraj

**Affiliations:** 1 College of Medicine, Imam Abdulrahman bin Faisal University, Dammam, SAU; 2 College of Medicine, Imam Abdulrahman Bin Faisal University, Dammam, SAU; 3 Emergency, King Fahad University Hospital, Dammam, SAU; 4 Emergency, Imam Abdulrahman Bin Faisal University, King Fahad University Hospital, Dammam, SAU

**Keywords:** hemodynamic instability, stab wound, bowel repair, penetrating trauma, abdominal trauma, jejunal perforation, peritonitis

## Abstract

Abdominal trauma injuries are caused by many mechanisms including blunt and penetrating trauma injuries. Penetrating injuries are far more common than blunt injuries. Subsequently, the most affected organ during such injuries is small intestine perforations. As far as we know, there were no cases reported before about jejunal injury due to penetrating trauma.

We present a case of a 20-year-old male with a stab wound injury who was initially stable in the emergency department (ED). After a set of investigations were done, the patient was discharged home. Yet, the patient presented again with late signs of peritonitis. Imaging was done and showed pneumoperitoneum. Thereafter, the patient was rushed to the operation room (OR) where the jejunal repair was performed. ED physicians must be vigilant regarding any signs of deterioration in penetrating trauma patients and should provide clear instructions to patients regarding any symptoms of the acute abdomen before any discharge.

## Introduction

One of the most prevalent causes of death worldwide is trauma, in particular, blunt injuries due to motor vehicle accidents [1]. To a lesser prevalence than the aforementioned, penetrating traumatic injuries are also a major cause of death and are a significant health burden in the world [2]. The mechanism of such trauma involves thrusting a sharp-edged object, where the force that is applied by the thrust produces a wound that is greater in depth and length than the weapon utilized [1]. Multiple articles reported that the most common cause of penetrating injuries is due to stabbing [1,2], which results in penetrating or perforating injuries. It is reported in some studies that the most common areas to attain a stab wound are the chest and abdomen, and in others, it is reported to be the upper extremities [1,3]. Ultimately, these injuries result in damage to the internal organs or vessels and can progress to infection, shock, and eventually death [1]. In one study, it was found that small bowel injuries were second to abdominal wall injuries [2].

In a study conducted in Jizan, Saudi Arabia, the mortality rate due to stab wounds were reported to be 7.3%. Thus, early interventions by conducting physical examinations focused on assessment with sonography in trauma (FAST), and local wound exploration (LWE) is of significant importance when it comes to managing these cases and assessing the risk of developing complications [4].

There is a clear consensus and no doubt that those patients who sustained a stab wound and are hemodynamically unstable indicate an immediate laparotomy. However, those who are evaluated to be stable are managed non-operatively. Non-operative management of stab wounds in stable patients has been long adapted to practice. Prior to the 1960s, it was common that such cases were managed surgically, were all traumatic penetrating wounds underwent operative exploration. This approach follows the basis and the reliability of clinical assessments only, where the patient is given analgesia, is closely observed and is frequently examined. An immediate exploratory laparotomy is indicated when the patient is seen to have an increase in abdominal tenderness, abdominal distention, tachycardia, or vomiting. This positive shift in management resulted in a decrease in the morbidity and mortality associated with these conditions [5]. The decision to order a computed tomography (CT) scan in these patients, however, is still not standardized in the literature. Some physicians do take this approach and order the imaging, while others rely on mere observation, examination, FAST, and LWE.

Therefore, our speculation lies in whether to observe such patients for a longer period in the ED or to discharge with clear instructions for all patients presenting to the ED with a penetrating stab wound who might present later with late signs of peritonitis.

## Case presentation

A 20-year-old Saudi male medically free presented to our ED after a flank stab wound two hours before presentation. Upon primary survey, the patient was hemodynamically stable, conscious, alert, and oriented with a Glasgow coma score of 15 out of 15. The airway was maintained with good equal air entry bilaterally. No signs of external bleeding were present. Pupils were reactive bilaterally and no spinal tenderness was observed. Upon imaging, FAST was done and showed no free fluid. While performing the secondary survey, a superficial wound over the left flank area measuring 3 cm with no active bleeding was found. Thereafter, the abdominal examination was performed, the abdomen was soft, lax, and not tender with no signs of peritonitis. systemic examination was unremarkable. The patient was managed with a wound suture of three stitches and the patient was discharged after two hours of observation with instructions to visit the ED whenever experiences abdominal pain, vomiting, change in bowel motion, or fatigue.

The patient presented again one day later in the ED complaining of severe, generalized abdominal pain, a burning sensation, radiating to the back that increases with movement and is associated with subjective fever. The patient stated that his appetite was decreased and coupled with nausea. According to the patient, there was a negative history of vomiting, hematemesis, diarrhea, or constipation. Upon examination, the patient was looking ill and in pain and distress. His vital signs were as the following: blood pressure of 125/74 mmHg, heart rate of 70 beats per minute, temperature 36.5 ºC, oxygen saturation of 99% on room air, and respiratory rate of 16 breaths per minute. On palpation of the abdomen, it was diffusely tender and rigid. The rest of the systemic examination was unremarkable. Furtherly, important investigations were ordered (complete blood cells, renal function test, liver function test, prothrombin time, activated partial thromboplastin time). All test results showed normal ranges except for the white blood cell count was high, up to 19.7 k/µL (normal: 4-11).

**Figure 1:**
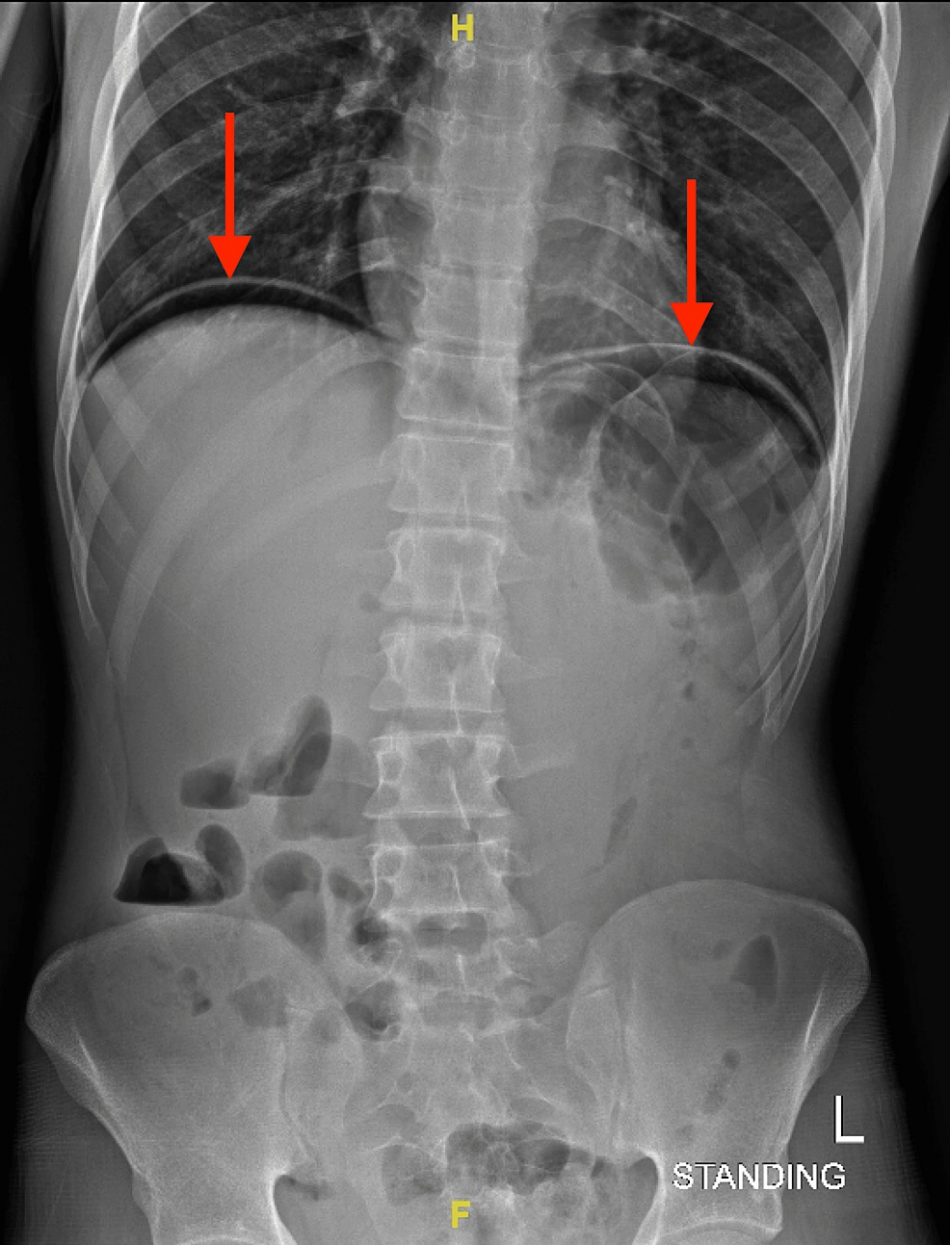
Erect abdominal x-ray. The red arrow shows bilateral air under the diaphragm.

**Figure 2:**
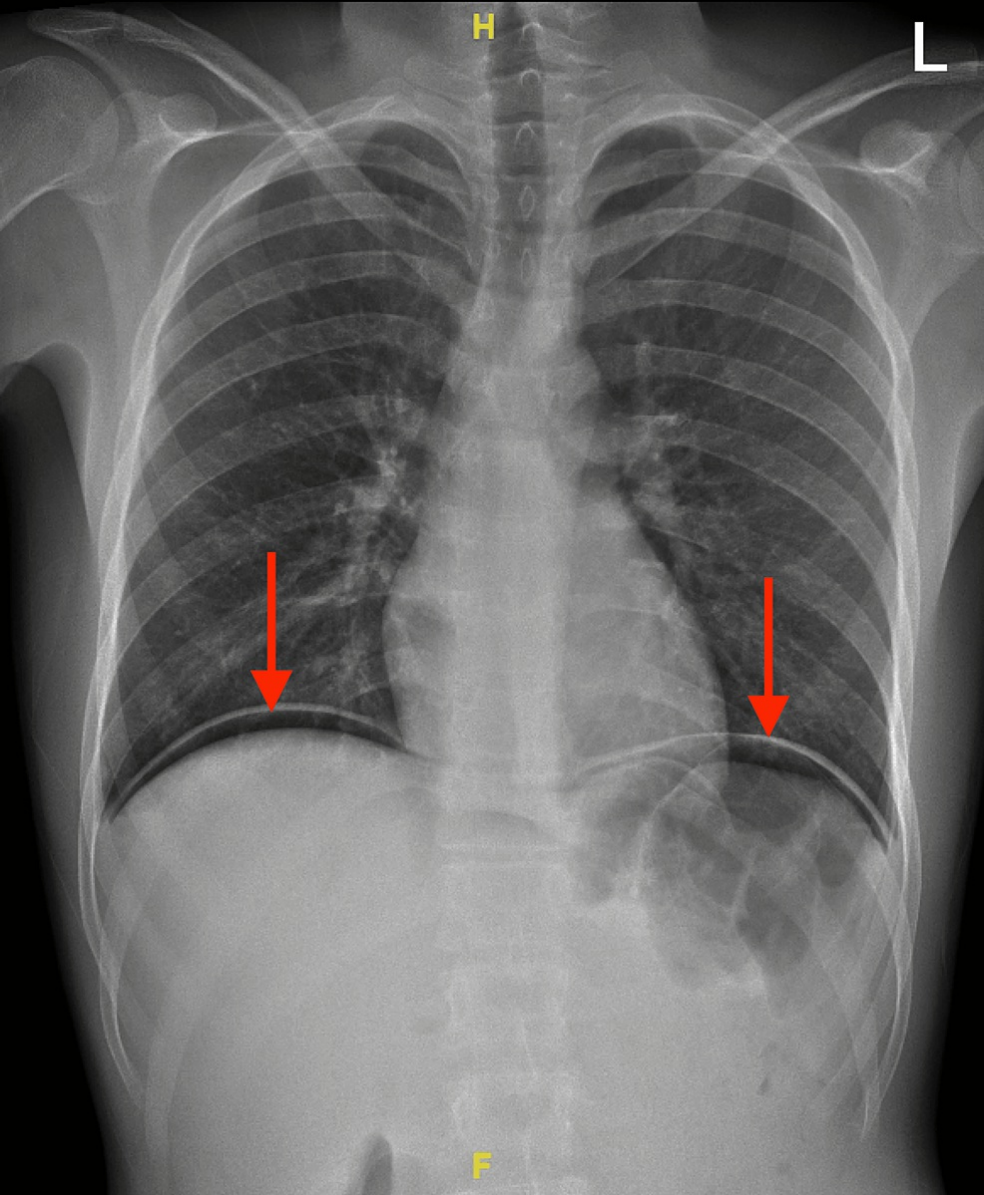
Posterior-anterior chest x-ray. The red arrow shows free gas under both diaphragmatic copula.

**Figure 3:**
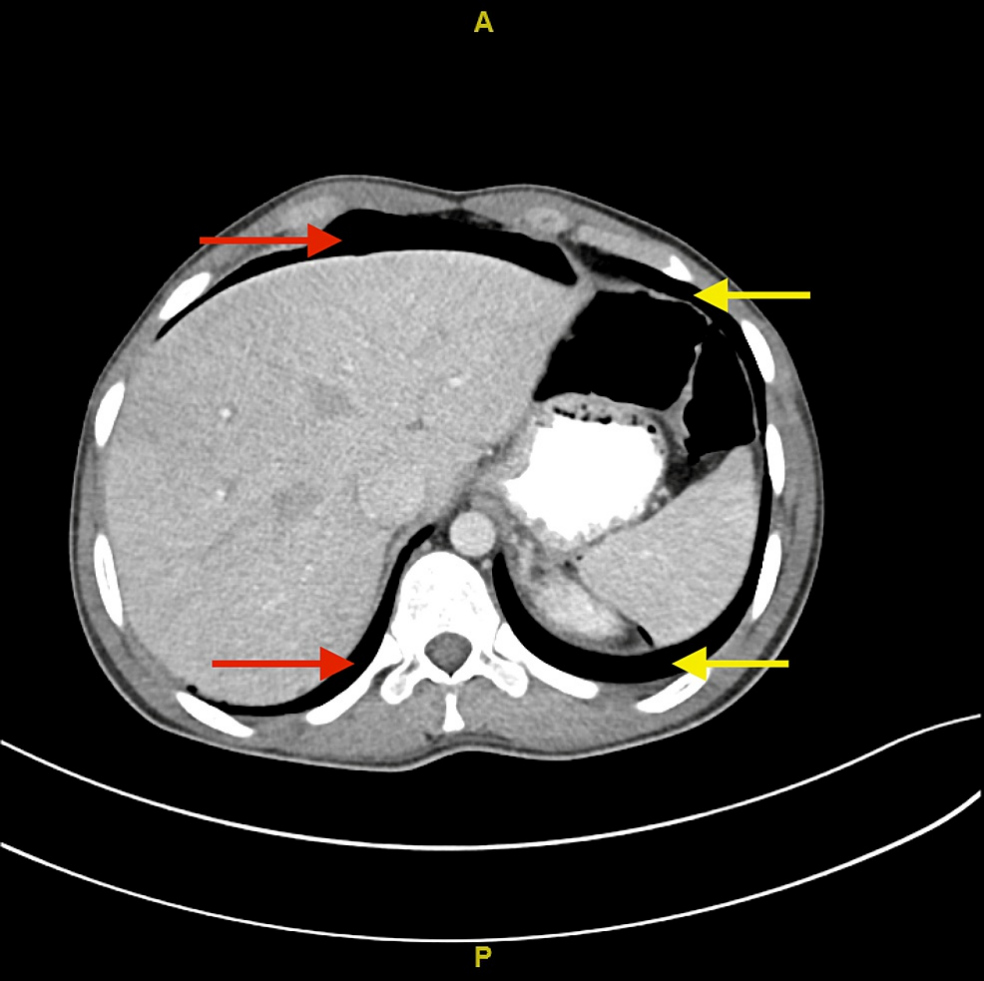
Axial CT of the abdomen. The red arrow shows air in the peri-hepatic area. The yellow arrow shows air in the peri-splenic area.

The patient was kept nothing by mouth and given ringer lactate intravenous (IV) fluids 1,000 mL. Erect abdominal X-ray and chest X-ray were performed and revealed air under the diaphragm (Figures 1, 2). CT scan with contrast (oral and IV) was later performed showing intra-abdominal pneumoperitoneum, and visceral bowel showed no wall enhancement defect or extravasation. However, a small visceral injury could not be ruled out (Figure 3). The patient was admitted to the hospital as a case of perforated viscus secondary to a stab wound in the OR. The patient underwent diagnostic laparoscopy. No free fluid was found, and the bowel looked intact. However, the surgeon decided to convert to laparotomy to achieve full visualization of the bowel. The jejunal injury was discovered, and small bowel repair was done. The patient’s operation went smoothly with no complications. Hence, the patient was transferred to the regular ward. On the third day of post-operative monitoring of the patient, his state indicated that the patient was in a good condition. Hereafter, the patient was discharged as he was tolerating a regular diet and passing flatus and stool.

## Discussion

Abdominal trauma injury is caused by many mechanisms including blunt and penetrating trauma injuries. Blunt injuries occur when there is an injury by an object with no open or broken skin. Penetrating injuries, on the other hand, occur when the object penetrates and breaks the skin and passes through the tissues. For instance, it could be due to gunshots and stab wounds. It can result in injuries to the intestine, liver, spleen, and rectum [6-9]. Penetrating injuries are more common than blunt injuries. In addition, the small intestine is the most affected organ followed by the liver (70.1% and 19.40%, respectively) [10].

Upon presentation in the ED, stab wound patients must be assessed with caution immediately. Resuscitation should be initiated in hemodynamically unstable patients [11]. Hollow viscus injuries must always be kept in mind in such a mechanism of injury. However, diagnosing visceral perforation can be challenging, leading to a delay in providing proper care. Hence, increasing morbidity and mortality rates [12]. Physicians should look for signs of peritonitis, which presents earlier in gastric perforation [13]. However, small intestine perforation tends to take a longer period for signs and symptoms to surface. The use of FAST scan in penetrating injury can detect hemoperitoneum [13]. Though, a negative scan does not warrant rolling out viscus injury. Furthermore, LWE should be done to check for peritoneal violations. The presence of positive FAST or LWE warrants a close clinical assessment including checking on vital signs, performing frequent physical examinations every four hours and checking on labs every eight hours to monitor any deterioration of the patient’s status [14]. Though, the presence of positive findings in both procedures does not indicate going for diagnostic laparoscopy. On the contrary, negative findings may permit the discharge of patients with clear instructions about their condition [14]. Nowadays, the use of triple contrast CT scans (oral, IV, and rectal contrast) in diagnosing perforated viscus is part of the practice in hemodynamically stable patients and carries 100% sensitivity in penetrating trauma [15]. On the other hand, the sensitivity, and specificity of CT scan ranges between 55%-95% and 48%-92%, respectively, in blunt trauma [16]. Nevertheless, CT scan use in hollow viscus injuries does not produce specific findings of bowel perforation, leading to difficulty for surgeons to decide on the need for operation [16]. Intraperitoneal fluid, solid organ injury, and pneumoperitoneum are signs for predicting hollow viscus injuries and aid in management [16]. In our case, FAST was performed upon the first presentation and showed no free fluid. However, in the second presentation, the x-ray showed air under the diaphragm. In addition, CT (oral and IV) was done and demonstrated pneumoperitoneum with no wall enhancement defect or extravasation.

The use of diagnostic laparoscopy is a debatable approach in hemodynamically stable patients. Some studies support the use of such an approach in the evidence of peritoneal penetration to decrease the rate of unnecessary laparotomies [16]. In contrast, other studies criticize the use of diagnostic laparoscopy in stable patients to decrease expenses, ineffectiveness, and morbidity [16]. Determining the approach to the management of hollow viscus injury is based on patient stability and serial clinical evaluations in the ED.

Managing intra-abdominal injuries is highly dependent on the presence of hemodynamical instability or signs of existing peritonitis [14]. Oyo-Ita et al. stated that in case of no evidence of internal bleeding or signs of peritonitis, the selective non-operative management (SNOM) approach is considered. SNOM consists of observing stable patients and allowing the healing process to act in its natural ways with frequent monitoring for any deterioration or indication for surgery. Non-therapeutic laparotomies number has been decreasing since the introduction of SNOM back in the 1960s, leading to shorter hospital stays, lower costs, and decreased mortality and morbidity [15]. However, the presence of evisceration, peritonitis, and hemodynamic instability all mandate emergent laparotomy regardless of the type of injury [15]. Small intestine perforation surgical management may be performed by simple primary repair, anastomosis, resection, or ostomy depending on the presenting case [17]. In our case, there was no indication for diagnostic laparoscopy as the patient was stable with a negative FAST scan. As a result, SNOM was our approach to managing our patients. After discharge, the patient presented with signs of late peritonitis which indicated an operative intervention for jejunal bowel repair.

Oyo-Ita et al. reviewed studies targeting the significant differences between observation versus operating in viscus injuries regarding morbidity and mortality. Based on the studies, there was no evidence of surgical management superiority over approaching patients with observation in the absence of peritonitis and bleeding [18]. In our case, the patient was kept for two hours of observation. During his stay in the ED, the patient did not show any signs of hemodynamic instability or signs of peritonitis. Nevertheless, the patient returned the other day after discharge with clear signs of peritonitis. Consequently, ED physicians must always bear in mind the probability of late peritonitis presentation in visceral injuries. To the best of our knowledge, no case reports were published regarding late peritonitis in similar cases. Therefore, we recommend giving trauma patients clear instructions in the ED regarding coming back if any acute abdomen symptoms arise before considering discharging them home to avoid late complications. Additionally, our patient had a jejunal perforation which was discovered in the OR. Interestingly, none of the penetrating trauma cases reported in the literature had a similar type of visceral perforation as in our case.

Therefore, in patients with penetrating injuries regardless of their hemodynamic stability and negative imaging, prolonged observation in ED or discharging the patient with clear instruction of signs of peritonitis should be considered to rule out late presentation of peritonitis to manage intraoperatively.

## Conclusions

Stab wound injuries are common and lead to various visceral injuries. Any intra-abdominal bleeding, bowel injury, or peritonitis should be ruled out by radiological investigations. However, negative radiological findings do not indicate the absence of the before-mentioned complications. Hence, in hemodynamically stable patients, clear instructions should be provided to avoid late complications. Thereafter, the decision to operate on such patients is a life-saving procedure.
